# Description of a Current Outbreak of *Mycoplasma pneumoniae* in the United States

**DOI:** 10.3390/pathogens14010060

**Published:** 2025-01-11

**Authors:** Anupama Raghuram, Stephen Furmanek, Thomas Chandler, Salwa Rashid, William Mattingly, Julio Ramirez

**Affiliations:** 1 Norton Infectious Diseases Institute, Norton Healthcare, Louisville, KY 40202, USA; anupama.raghuram@nortonhealthcare.org (A.R.); stephen.furmanek@nortonhealthcare.org (S.F.); thomas.chandler@nortonhealthcare.org (T.C.); salwa.rashid@nortonhealthcare.org (S.R.); william.mattingly@nortonhealthcare.org (W.M.); 2 Division of Infectious Diseases, University of Louisville, Louisville, KY 40202, USA

**Keywords:** *Mycoplasma pneumoniae*, respiratory illness, disease surveillance, pneumonia

## Abstract

During the COVID-19 pandemic, a significant decline in *Mycoplasma pneumoniae* was observed; however, *M. pneumoniae* re-emerged globally in 2023. Here, we describe a current outbreak of *M. pneumoniae* infections in the United States (US). More than 287 million patient records from all 50 states in the US were reviewed to identify patients with a *M. pneumoniae* diagnosis between 1 January 2017 and 30 September 2024. A c-chart was created by calculating the mean and standard error (SE) of cases during the pre-COVID-19 pandemic period, with the upper control limit (UCL) set at 3 SE above the mean. The presence of an outbreak was defined as counts above the UCL. Cumulative excess cases were used to estimate the magnitude of the outbreak, and the fold increase was calculated. A US outbreak of *M. pneumoniae* began at the end of 2023, resulting in 9708 excess cases corresponding to a 9.0-fold increase over the baseline UCL. The outbreak is ongoing, affects both children and adults, and includes patients with *M. pneumoniae* community-acquired pneumonia requiring hospitalization. This US outbreak of *M. pneumoniae* has significant implications for the management of patients with respiratory infections during the current pneumonia season.

## 1. Introduction

*Mycoplasma pneumoniae* is a well-recognized respiratory pathogen affecting children, adolescents, and adults that can cause community-acquired pneumonia severe enough to require hospitalization [1,2]. The organism has a slow incubation period ranging from one to four weeks and is associated with cyclic epidemics occurring every three to five years [3,4]. This extended incubation period may contribute to prolonged outbreaks, allowing the infection to spread undetected over time. The cyclical nature of these regular *M. pneumoniae* outbreaks is likely influenced by changes in population immunity. Following an outbreak, a significant proportion of those infected typically develops immunity, temporarily reducing the number of susceptible individuals and decreasing the potential for widespread transmission until immunity in the population wanes.

Several studies have observed a decrease in *M. pneumoniae* infections during the COVID-19 pandemic, likely due to public health measures such as masking, physical distancing, and reduced social interactions. Globally, a subsequent rebound in the number of infections to the pre-pandemic levels beginning in 2023 has been observed [5,6,7]. Local *M. pneumoniae* outbreaks have been reported in France and Switzerland [8,9,10]. In the United States, the Centers for Disease Control and Prevention (CDC) reported an increase in *M. pneumoniae* infections among children and adolescents starting in the fall of 2023, although the number of cases remained below the pre-COVID-19 pandemic levels [11]. Additionally, in October 2024, the CDC issued an alert regarding an increase in the percentage of *M. pneumoniae* testing positivity across all age groups nationwide, with the most significant increase observed among children [12].

In the city of Louisville, Kentucky, we identified an increase in hospitalized adult patients with *M. pneumoniae* as part of an ongoing local respiratory infection surveillance study. These local findings prompted us to analyze national data with the goal of defining whether the local increase reflects a broader outbreak of *M. pneumoniae* infections within the United States.

## 2. Materials and Methods

This was a retrospective analysis of the United States Epic Cosmos database [13,14]. Epic Cosmos is a de-identified research database created in collaboration with a community of Epic health systems representing over 287 million patient records from over 1600 hospitals and 36,000 clinics from all 50 states in the United States and Washington D.C. as of the study period. More than 11 billion patient interactions with an Epic-using medical facility (encounters such as a clinic visit, emergency department visit, or hospitalization) from 1 January 2017 to 30 September 2024 were queried for analysis.

A patient was defined as having a *M. pneumoniae* infection if they met the following criteria: (1) they had a patient encounter at an Epic-using facility in the United States, and (2) they had a positive nucleic acid amplification test (NAAT) for *M. pneumoniae* during that encounter. A positive test for *M. pneumoniae* was identified using specified Logical Observation Identifiers Names and Codes (LOINC) [14] (see Table S1 in the Supplementary Materials).

Patients with encounters during the timeframe of 1 January 2017 to 30 September 2024 were included in this analysis. Infections documented before the emergence of the COVID-19 pandemic were defined as baseline infections from the timeframe of 1 January 2017 to 31 March 2020. Infections documented after the COVID-19 pandemic were defined as current infections from the timeframe of 1 July 2022 to 30 September 2024.

To identify the presence of an outbreak, all patients with *M. pneumoniae* were aggregated in annual quarters and depicted in count charts (c-charts) [15]. Additionally, c-charts were created for pediatric (age < 18) and adult (age ≥ 18) patients. Expected control limits were derived from the baseline infection period. The mean number of monthly cases (µ) and standard error (SE) were calculated for the baseline infections, with the upper control limit (UCL) set at 3 SE above the mean. The presence of an outbreak was defined as any quarter above the baseline UCL.

The number of excess cases for each annual quarter, if any, was calculated by taking the number of cases in excess of the baseline UCL. Cumulative excess cases for all patients, pediatric patients, and adult patients were calculated and depicted in line graphs. Additionally, excess cases for patients hospitalized with *M. pneumoniae* pneumonia were calculated. Cumulative excess cases of all patients, pediatric patients, and patients hospitalized with *M. pneumoniae* pneumonia were also depicted in line graphs. The magnitude of excess cases was defined as the fold increase in excess cases above the baseline UCL for the quarter with the highest excess.

### Statistical Analysis

Patient demographics of age, sex, race, and ethnicity were compared between current infections and baseline infections. The severity of disease for baseline and current infections was measured by (1) the number of patients hospitalized with pneumonia, as defined by an admission using the tenth International Classification of Diseases (ICD-10) codes to define pneumonia; (2) the number of patients requiring mechanical ventilation, defined by Current Procedural Terminology (CPT) codes; and (3) the number of patients who died, defined by the Epic-reported disposition (see Tables S2 and S3 in the Supplementary Materials for ICD-10 and CPT codes).

C-chart epidemic curves and line graphs were produced to visualize the data. Data were aggregated by quarter. Baseline and current patient characteristics and the severity of disease were compared using Chi-Squared tests of independence. Groups with less than or equal to 10 observations were censored as ≤10 to prevent the identification of data. A *p*-value of less than 0.05 was considered statistically significant. All data analyses were performed using R version 4.4.2.

## 3. Results

**Figure 1:**
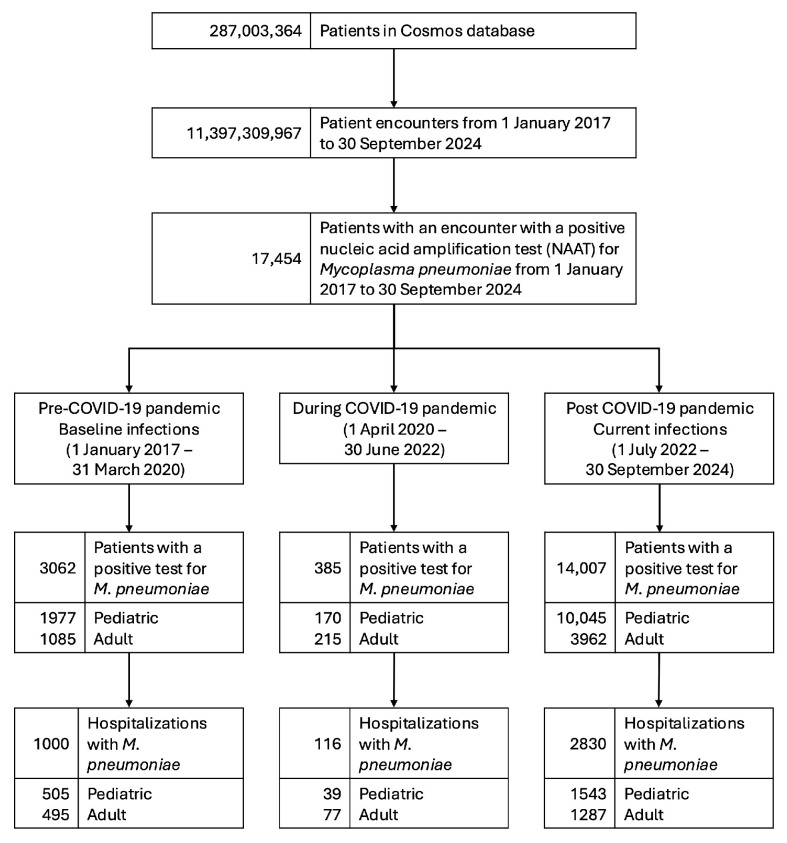
Study flowchart detailing patients with *M. pneumoniae* infection.

A total of 17,454 patients with *M. pneumoniae* infection were identified, with 3062 during the baseline period, and 14,007 during the current period. A total of 3946 patients were hospitalized with *M. pneumoniae* pneumonia, with 1000 patients hospitalized during the baseline period and 2830 hospitalized during the current period. During the COVID-19 pandemic, 385 patients with *M. pneumoniae* infection were observed, with 116 hospitalized. The study flow diagram is depicted in Figure 1.

**Figure 2:**
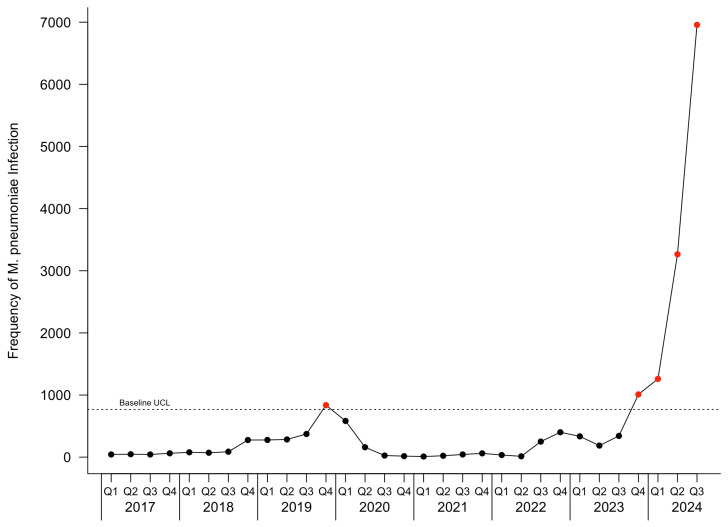
Epidemic curve c-chart for all patients with *M. pneumoniae* infection. The baseline upper control limit (UCL) is depicted by the dotted horizontal line. Points colored black represent frequencies within the UCL, and points in excess of the UCL are colored red.

**Figure 3:**
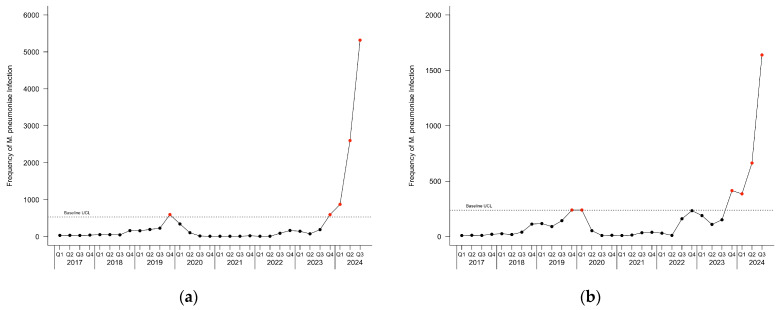
Epidemic curve c-charts for (**a**) pediatric patients and (**b**) adult patients with *M. pneumoniae* infection. The baseline upper control limit (UCL) is depicted by the dotted horizontal line. Points colored black represent frequencies within the UCL, and points in excess of the UCL are colored red.

The c-chart for patients with *M. pneumoniae* infection is depicted in Figure 2. Figure 3 depicts the c-charts for pediatric and adult patients with *M. pneumoniae* infection. During the baseline period, one point was observed above the UCL in the fourth quarter of 2019 for all patients and pediatric patients, and two points were observed above the UCL in the fourth quarter of 2019 and the first quarter of 2020 in adult patients. During the current infection period, an outbreak started in the fourth quarter of 2023 and continued through the last observed quarter.

**Figure 4:**
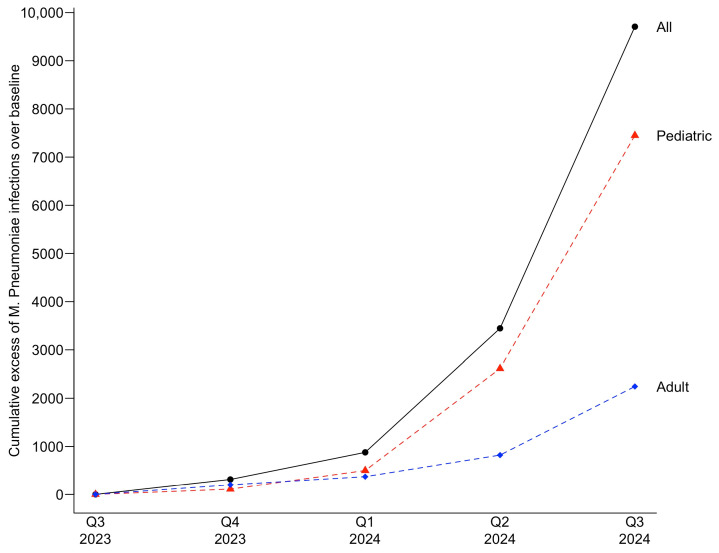
Cumulative excess cases of *M. pneumoniae* infections during the current infection period.

The cumulative excess case count for the current infection period was 9708 for all patients. The highest increase above the UCL, representing a 9.0-fold increase, was observed in the third quarter of 2024, the last quarter evaluated in the current infection period in this study. For pediatric patients, the cumulative excess was 7450, with a 10.0-fold increase over the baseline UCL observed in the third quarter of 2024. For adults, the cumulative excess was 2264, with a 6.6-fold increase over the baseline UCL also in the third quarter of 2024. Figure 4 depicts the cumulative excess cases for the current infection period.

**Figure 5:**
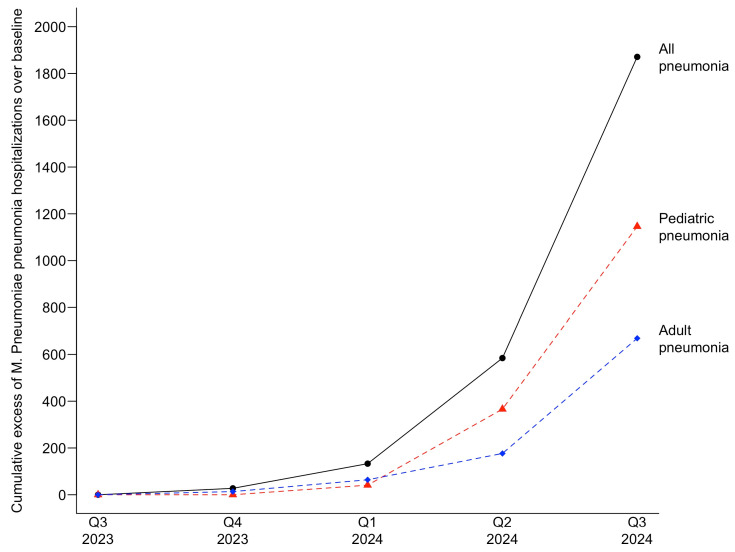
Cumulative excess cases of patients hospitalized with *M. pneumoniae* pneumonia in the current infection period.

Excess cases of patients hospitalized with *M. pneumoniae* pneumonia were also observed for the current infection period. Cumulative excess cases of 1870 for all patients, 1147 for pediatric patients, and 670 for adult patients, with a 7.7-fold, 8.6-fold, and 5.4-fold increase over the baseline UCL, respectively, were observed in the third quarter of 2024. Figure 5 depicts the cumulative excess cases of patients hospitalized with *M. pneumoniae* pneumonia for the current infection period.

Pediatric and adult patient demographics comparing baseline and current infection periods are depicted in Table 1. Overall, patient characteristics for both populations were similar between baseline and current infection periods.

The severity of disease for all patients is depicted in Table 2. A smaller proportion of patients with *M. pneumoniae* were hospitalized during the current infection period. The need for mechanical ventilation and the rate of death were similar among baseline and current infection periods in adults, and were infrequently observed in pediatric patients.

## 4. Discussion

This study provides evidence of an ongoing outbreak of *M. pneumoniae* infections in the United States that began in late 2023 and is affecting both pediatric and adult populations. To our knowledge, this is the first report of the current outbreak in the United States. Given the absence of a plateau in the number of new cases, it is likely that this outbreak will extend into the 2024–2025 winter pneumonia season. Analysis of the outbreak, including the number and magnitude of excess cases, indicates that the pediatric population is disproportionally more affected compared to the adult population.

Data from our study suggest that a regular cyclic outbreak of *M. pneumoniae* was developing in the United States by late 2019 but was abruptly interrupted by the occurrence of the COVID-19 pandemic. This interruption was likely due to the introduction of non-pharmacological interventions against COVID-19. Similar outbreaks of *M. pneumoniae* were also reported in Europe and Asia at the end of 2019 [16]. We observed that detection of *M. pneumoniae* in the United States decreased significantly during the period of the COVID-19 pandemic. A decrease in other bacterial respiratory pathogens such as *Streptococcus pneumoniae* and *Haemophilus influenzae* was also reported during the COVID-19 pandemic period [17]. This decline in infections likely resulted in decreased global population immunity, increasing susceptibility to *M. pneumoniae* as COVID-19-related restrictions were lifted. Although our data document an ongoing *M. pneumoniae* outbreak in the United States, it is possible that similar outbreaks are occurring in other regions with limited surveillance capabilities, leading to underdiagnosis on a global scale.

Aside from age, similar demographic characteristics were observed in patients with *M. pneumoniae* infection in the current and baseline periods, suggesting that the current outbreak is affecting a similar patient population. Unexpectedly, we did not document an increase in the severity of disease for *M. pneumoniae* infections in the current outbreak considering the lack of increase in the number of patients with pneumonia requiring hospitalization, the number of patients with need for mechanical ventilation, and the number of deaths compared to the pre-COVID-19 baseline period. These findings could be due to increased proportions of ambulatory testing using NAAT detection for respiratory pathogens that may have skewed the number of patients hospitalized.

The current outbreak is associated with a significant increase in cases of *M. pneumoniae* community-acquired pneumonia, with patients of all ages requiring hospitalization for care. This resurgence has several implications for clinical practice: (1) Clinicians should maintain a high index of suspicion for *M. pneumoniae* as a cause of respiratory infections, particularly in patients presenting with community-acquired pneumonia; (2) The diagnosis of *M. pneumoniae* infections is challenging since the organism is not detectable on Gram stain—due to the lack of a cell wall—and does not grow from respiratory or blood samples using standard culture methods. The primary method of diagnosis is NAAT to detect the genetic material of the organism from respiratory specimens; (3) *M. pneumoniae* is intrinsically resistant to all beta-lactam antibiotics because it lacks a cell wall, the target for beta-lactam bactericidal activity. Consequently, empiric monotherapy with beta-lactams is ineffective against this pathogen; (4) The possibility of macrolide-resistant *M. pneumoniae* in hospitalized patients with pneumonia who fail to respond to macrolide therapy should be considered. Macrolide resistance in the US varies regionally, with reported rates between 2% and 22% [12]. Alternatives for treating macrolide-resistant *M. pneumoniae* include tetracyclines and fluoroquinolones as clinical isolates with acquired resistance to these antibiotics have not been reported [18]; (5) clinicians should remain vigilant about the range of possible extrapulmonary manifestations of *M. pneumoniae* infections such as dermatological, cardiovascular, or neurological complications. These manifestations may be present in patients with minimal or absent respiratory symptoms [19].

This study has several strengths, including the capability to evaluate more than 280 million patients from across the United States, the ability to obtain data on *M. pneumoniae* infections before the COVID-19 pandemic to define a pre-pandemic baseline level of infections for comparison, the inclusion of patients with diagnosis based solely on positive NAAT of respiratory samples, and the capability to analyze subpopulations such as children and adults, with and without *M. pneumoniae* community-acquired pneumonia requiring hospitalization. Since the Epic Cosmos database is standardized, reflects real-world clinical practice, and is representative of a large proportion of the United States population, our findings are likely highly generalizable across the country.

The primary limitations of this study include the lack of data on the total number of diagnostic tests ordered for *M. pneumoniae* infections, which did not allow us to calculate the percentage of positive tests. Additionally, in this analysis, we considered every positive test as a case of *M. pneumoniae* infection; therefore, if a patient had a positive test in a clinic and later required hospitalization for the same infection and was tested an additional time, while rare, this case would have been counted as two infections. Lastly, we were unable to assess the incidence of *M. pneumoniae* co-infections with other viral or bacterial respiratory pathogens.

## 5. Conclusions

In conclusion, this study documents the presence of an ongoing outbreak of *M. pneumoniae* infections across the United States affecting both pediatric and adult populations. The current outbreak has significant implications for the management of patients with respiratory infections during the 2024–2025 winter pneumonia season. Continued surveillance at a national and global level is necessary to define the extent and clinical impact of this current *M. pneumoniae* outbreak.

## Figures and Tables

**Table 1:** Demographic comparison of baseline and current patients with *M. pneumoniae* infection.

Patient Demographics	Baseline (*n* = 3062)	Current (*n* = 14,007)	*p*-Value
Age			
<1 years	149 (4.9)	426 (3.0)	<0.001
1–4 years	563 (18.4)	2448 (17.5)	0.242
5–9 years	636 (20.8)	3604 (25.7)	<0.001
10–17 years	629 (20.5)	3578 (25.5)	<0.001
18–44 years	576 (18.8)	2260 (16.1)	<0.001
45–64 years	279 (9.1)	868 (6.2)	<0.001
65–84 years	200 (6.5)	719 (5.1)	0.002
≥85 years	30 (1.0)	104 (0.7)	0.217
Sex at birth			
Male	1609 (52.5)	7332 (52.3)	0.855
Female	1453 (47.5)	6675 (47.7)	0.855
Race and ethnicity ^1^			
White race	2397 (78.3)	10,468 (74.7)	<0.001
Black race	346 (11.3)	1856 (13.3)	0.004
Asian race	107 (3.5)	474 (3.4)	0.802
American Indian race	41 (1.3)	94 (0.7)	<0.001
Native Hawaiian race	20 (0.7)	74 (0.5)	0.477
Other race	405 (13.2)	2354 (16.8)	<0.001
Hispanic	653 (21.3)	2508 (17.9)	<0.001

^1^ Race and ethnicity were not mutually exclusive and may add up to more than 100%.

**Table 2:** Severity of disease for baseline and current patients with *M. pneumoniae* infection.

Severity of Disease	Pediatric (Age < 18 Years)			Adult (Age ≥ 18 Years)		
	Baseline (n = 1977)	Current (n = 10,045)	*p*	Baseline (n = 1085)	Current (3962)	*p*
Hospitalization with pneumonia, n (%)	505 (25.5)	1543 (15.4)	<0.001	495 (45.6)	1287 (32.5)	<0.001
Need for mechanical ventilation, n (%)	≤10 (*)	≤10 (*)	- ^1^	35 (3.2)	87 (2.2)	0.067
Death, n (%)	≤10 (*)	≤10 (*)	- ^1^	≤10 (*)	23 (0.6)	- ^1^

* Percentage not reported due to suppressed data in order to maintain patient confidentiality. ^1^ Comparison not performed due to suppressed data in order to maintain patient confidentiality.

## Data Availability

The original contributions presented in this study are included in the article. Further inquiries can be directed to the corresponding author.

## Supplementary Materials

The following supporting information can be downloaded at https://www.mdpi.com/article/10.3390/pathogens14010060/s1, Table S1: Logical Observation Identifiers Names and Codes (LOINC) queried to identify the presence of *M. pneumoniae* infection; Table S2: International Classification of Diseases, Tenth Revision (ICD-10) codes queried to identify encounters pneumonia; Table S3: Study flowchart of patients with *M. pneumoniae* infections in the Cosmos database; Table S4: Rate of *M. pneumoniae* infection by US State or Territory.

## Abbreviations

The following abbreviations are used in this manuscript:

COVID-19	Coronavirus disease of 2019
SE	Standard error
UCL	Upper confidence limit
NAAT	Nucleic acid amplification test
LOINC	Logical Observation Identifiers Names and Codes
ICD-10	Tenth International Classification of Diseases
CPT	Current Procedural Terminology
