# Supplementary material for: Description of a Current Outbreak of Mycoplasma pneumoniae in the United States
Source: Pathogens. 2025 Jan 11;14(1):60. doi: 10.3390/pathogens14010060 (PMC11768315; doi:10.3390/pathogens14010060)
Supplement: Supplementary file 1 [file pathogens-14-00060-s001.zip › pathogens-3413078-supplementary.pdf]

Supplemental materials for *Description of a Current Outbreak of Mycoplasma Pneumonia in United States*

Authors: Anupama Raghuram, Stephen Furmanek, Thomas Chandler, Salwa Rashid, William Mattingly, Julio Ramirez

**Table of Contents**	
**Title**	**Page**
**Table S1**. Logical Observation Identifiers Names and Codes (LOINC) queried to identify the presence of *M. pneumoniae* infection	3
**Table S2**. International Classification of Diseases, Tenth Revision (ICD-10) codes queried to identify encounters pneumonia	3
**Table S3**. Current Procedural Terminology (CPT) codes used to identify encounters that required emergency intubation, mechanical ventilation, and critical care	3
**Table S4**. Rate of *M. pneumoniae* infection by US State or Territory	4

Table S1. LOINC queried to identify the presence of *M. pneumoniae* infection

LOINC	Specimen Type	Description
82177-7	Nasopharyngeal swab	Nucleic acid amplification with non-probe detection
88720-8	Nasopharyngeal swab	Nucleic acid amplification with probe detection
91804-5	Nasopharyngeal swab, oropharyngeal swab	Nucleic acid amplification with probe detection
92126-2	Respiratory	Nucleic acid amplification with probe detection

Table S2. ICD-10 codes queried to identify patients with pneumonia

ICD-10	Description
J12	Viral pneumonia, not elsewhere classified
J13	Pneumonia due to Streptococcus pneumoniae
J14	Pneumonia due to Hemophilus influenzae
J15	Bacterial pneumonia, not elsewhere classified
J16	Pneumonia due to other infectious organisms, not classified elsewhere
J17	Pneumonia in diseases classified elsewhere
J18	Pneumonia, unspecified organisms

Table S3. CPT codes used to identify encounters that required emergency intubation, mechanical ventilation, critical care

Procedure	CPT
Endotracheal Intubation (Emergency Procedure)	31500
Ventilation assist and management, initiation of pressure or volume preset ventilators for assisted or controlled breathing; hospital inpatient/observation, initial day.	94002
Ventilation assist and management, initiation of pressure or volume preset ventilators for assisted or controlled breathing; hospital inpatient/observation, subsequent days.	94003
Under Ventilator Management	94004
	94005

Table S4. Rate of *M. pneumoniae* infection by US State or Territory

State or Territory	2020 Census Population	Number of *M. pneumoniae* infections	Rate per 100,000 population
Alabama	5,024,279	77	1.53
Alaska	733,391	≤10	*
Arizona	7,151,502	36	0.5
Arkansas	3,011,524	613	20.36
California	39,538,223	172	0.44
Colorado	5,773,714	465	8.05
Connecticut	3,605,944	135	3.74
Delaware	989,948	78	7.88
District of Columbia	689,545	≤10	*
Florida	21,538,187	2726	12.66
Georgia	10,711,908	490	4.57
Hawaii	1,455,271	≤10	*
Idaho	1,839,106	≤10	*
Illinois	12,812,508	561	4.38
Indiana	6,785,528	188	2.77
Iowa	3,190,369	688	21.56
Kansas	2,937,880	≤10	*
Kentucky	4,505,836	512	11.36
Louisiana	4,657,757	62	1.33
Maine	1,362,359	≤10	*
Maryland	6,177,224	295	4.78
Massachusetts	7,029,917	26	0.37
Michigan	10,077,331	57	0.57
Minnesota	5,706,494	45	0.79
Mississippi	2,961,279	507	17.12
Missouri	6,154,913	149	2.42
Montana	1,084,225	33	3.04
Nebraska	1,961,504	82	4.18
Nevada	3,104,614	36	1.16
New Hampshire	1,377,529	58	4.21
New Jersey	9,288,994	312	3.36
New Mexico	2,117,522	≤10	*
New York	20,201,249	371	1.84
North Carolina	10,439,388	547	5.24
North Dakota	779,094	22	2.82

Ohio	11,799,448	364	3.08
Oklahoma	3,959,353	141	3.56
Oregon	4,237,256	51	1.2
Pennsylvania	13,002,700	244	1.88
Rhode Island	1,097,379	≤10	*
South Carolina	5,118,425	2295	44.84
South Dakota	886,667	18	2.03
Tennessee	6,910,840	285	4.12
Texas	29,145,505	539	1.85
Utah	3,271,616	25	0.76
Vermont	643,077	≤10	*
Virginia	8,631,393	128	1.48
Washington	7,705,281	74	0.96
West Virginia	1,793,716	308	17.17
Wisconsin	5,893,718	144	2.44
Wyoming	576,851	25	4.33

*Rate not calculated due to suppression of results
